# The feasibility of sodium hydroxide pretreatment of rice straw for solid substrate preparation to enhance laccase production by solid state fermentation

**DOI:** 10.1186/s12896-023-00789-3

**Published:** 2023-06-30

**Authors:** Lulu Wang, Ni An, Junting Gao, Huiting Xue, Guanhua Li

**Affiliations:** 1 State Key Laboratory of Reproductive Regulation and Breeding of Grassland Livestock, School of Life Sciences, Inner Mongolia University, Hohhot, 010070 China; 2 College of Basic Medicine, Inner Mongolia Medical University, Hohhot, 010110 China

**Keywords:** Cellulosic substrate, Rice straw, Solid state fermentation, Pretreatment, Laccase, Fermentability

## Abstract

**Background:**

Currently, broad industrial application of laccases is commonly restricted by the high-cost related production. Solid state fermentation (SSF) using agricultural waste is an attractively economic strategy for laccase production, yet its efficiency is low. Pretreatment of cellulosic substrate might be a vital breakpoint to solve the problem in solid state fermentation (SSF). In this study, sodium hydroxide pretreatment was involved to prepare solid substrates from rice straw. Fermentability of solid substrates in terms of carbon resource supply, accessibility and water retention value, and their influence on performance of SSF were analyzed.

**Results:**

The results showed that sodium hydroxide pretreatment provided desirable solid substrates with higher enzymatic digestibility and optimal water retention value, which further facilitated the homogeneity of mycelium growth, laccase distribution and nutrition utilization during SSF. The pretreated rice straw (1 h) with diameter less than 0.085 cm gave the maximum laccase production of 2912.34 U/g, which was 7.72 times higher than the control.

**Conclusion:**

Hence, we proposed that enough balance between nutrition accessibility and structure support was a must for rational design and preparation of solid substrate. Additionally, sodium hydroxide pretreatment of lignocellulosic waste might be an ideal step to enhance the efficiency and lower the production cost in SSF.

**Supplementary Information:**

The online version contains supplementary material available at 10.1186/s12896-023-00789-3.

## Introduction

Rice, with an annual production of around 700 million Tons, is the second most consumed food in the globe. It is estimated that more than 980 million Tons of rice straw is generated every year [1]. Biorefinery platform via enzymes for generation of industrially available commodities from these lignocellulosic wastes are consistent with the new paradigm of a circular, sustainable and low-carbon bioeconomy [2]. Lignocellulolytic enzymes take up more than 20% of sales of commercial enzyme around the worldwide [3]. Laccases (EC.1.10.3.2, benzenediol: O_2_ oxidoreductase) widely distribute in fungi, bacteria, plants and insects, and have been overproduced by genetically modified strains [4]. Laccases belong to blue multicopper oxidase families and exhibit distinctly non-specific substrate. Four copper ions at active site are implicated in electrons transfer from anilines, aromatic thiols and substituted phenols to O_2_. Thus, laccases show huge potential in the application of bio-pulping, biofuels production and lignin valorization, whereas, the techno-economic analysis suggests that more efforts are required to lower the production cost [5].

Both solid state fermentation (SSF) and submerged fermentation (SF) are the commonly used technology for laccase production [6, 7]. SSF with higher product yields and nearly no waste water, etc. is superior to SF [8]. Liquid as continuous film is attached on the solid substrate. Gas rather than liquid is the primary mobile phase in SSF, which leads to the difficulty in mass and heat transfer [9]. Additionally, the components in SSF exhibit high heterogeneity. Culture mediums typically prepared as standardized formulations in SF seem improper for SSF [10]. Thus, there are the major challenges encountered during the development of large-scale SSF. As the core component in SSF, solid substrate determines the related morphology, physiology and gene expression of microbes [11]. Thus, solid substrate is the key variable that cannot be circumvented for bioprocess design, parameter optimization and process explanation. Nevertheless, the impacts of physicochemical properties of solid substrate on SSF performance are still ambiguous.

Lignocellulosic waste, mainly composing of cellulose, hemicelluloses and lignin, is considered as the abundant, bio-degradable and renewable resource on the earth. Lignocellulosic waste can serve as a fixed support, nutrition resource and inducer, which has been broadly applied to lignocellulolytic enzyme production through SSF, especially for laccase, cellulase, xylanase, protease and pectinase [12]. However, complicated composition and heterogeneous integration structure of lignocellulosic waste restrict nutrition availability and heat and mass transfer, particularly for the inner region. Our previous research suggests that alkaline pretreatment of solid substrate could solve this problem [9]. On this premise, it is highly requiring users to treat the solid substrates prior to fermentation and determine the relationship between parameters of solid substrate and fermentation efficiency. Sodium hydroxide pretreatment requires less caustic reagent and milder conditions than other pretreatments, which reduce the demand for expensive materials and special designs [13]. Yet, there is less reports concerning the utilization of sodium hydroxide pretreatment in SSF.

In this feasibility study, alkaline pretreatment with sodium hydroxide as an active reagent was used to prepare solid substrates from rice straw. The effects of physicochemical properties of solid substrates on laccase production were analyzed. The distributions of mycelium and laccase on solid substrates were also observed through antibody labeling and porosity analysis. This research will enrich the theory of SSF and provide vital technical basis for laccase production.

## Materials and methods

### Materials and regents

Rice straw was kindly gifted by the local farmer in Xinganmeng, China. The primary antibody for laccase was prepared by ABcolnal Biotechnology (China). Cellulase (Batch number: 9012-54-8) from *Trichoderma viride* G was bought from Shanghai Yuanye Bio-Technology Co. Ltd. Secondary antibody (Mouse Anti-rabbit IgG/HRP), tissue freezing medium (4 fl.), polyvinylidene difluoride membrane (PDM), and 2, 2-Azinobis (3-ethylbenzo-thiazoline-6-sulfonic acid) (ABTS) were bought from Shanghai Yeyuan (China), Solarbio (China), Leica (USA), and Bio-Rad (Germany), Sangon Biotech (China), respectively. Other analytical reagents were purchased from Huhhot Shengkang Biotechnology Company (China).

### Strain collection and maintenance

*Funalia trogii* IFP0027 was acquired from the Culture Collection Center of the Institute of Shenyang Applied Ecology, Chinese Academy of Sciences, stored at 4 °C, and routinely subcultured every month.

### Preparation of solid substrates

Rice straw with length of around 3 cm was pretreated by 4% (w/v) NaOH solution at a solid loading of 5% to prepare pretreated solid substrate (PSS). This process was performed in screw-capped bottles with a working volume of 150 mL at 70 °C for 0.5, 1 and 1.5 h, respectively. After that, the remaining residues were filtered and washed thoroughly by distilled water to neutral pH. The PSS was oven dried at 65 °C to a constant weight and separated by hand. The raw rice straw was used as blank control (CK) in fellow study. The CK and PSS were divided into two fractions. One fraction was cut into small pieces with size around 2.5, 1.5, 0.5 cm in length. The other fraction was ground and passed through sieve to obtain powder with size arranging from 0.84 mm to 0.42 mm in length.

### Laccase production by solid state fermentation

Before SSF the stored strain was activated on malt extract agar plate (20 g/L malt extract, 18 g/L agar and pH 5) with an aerobic atmosphere at 28 °C for 120 h according to our previous reports [14]. Three mycelium agar disks with diameter of 6 mm were excised from edge of actively extending fungal mycelium and transferred into liquid culture medium (2 g/L soluble starch, 3 g/L peptone, 0.5 g/L copper sulfate and pH 5). The culture medium was kept in a rotary shaker with an aerobic atmosphere at 180 rpm and 28 °C for 36 h to prepare inoculums. SSF were performed in packed-bed glass columns with an internal diameter of 4 cm and a height of 20 cm. Solid substrates (CK and PSS) were mixed with liquid culture medium (2 g/L soluble starch, 3 g/L peptone, 0.5 g/L copper sulfate and pH 5; 3 mL per gram dry solid substrate), filled in the glass columns till the maximum packed density, and sterilized at 121 °C for 35 min in autoclave. The cooled medium was inoculated with inoculums (0.5 mL per gram dry solid substrate) and gently agitated to ensure the homogeneous distribution of mycelium on solid substrate. SSF was performed with an aerobic atmosphere, at 28 °C for 14 d.

### Characterization of solid substrate

Various powder samples with size arranging from 0.84 mm to 0.42 mm in length were selected to characterize physicochemical properties. Chemical composition was determined according to Van Soest protocol [15]. Water retention value (WRV) was assayed by a centrifugal method [16]. Cellulose accessibility (CA) was simulated by a Langmuir adsorption isotherm of Congo red and calculated with Eq. 1 [17]. Enzymatic digestibility (ED) was evaluated through in vitro cellulase mediated digestion [18]. Specific surface area (SSA) and porous parameters were assayed by Brunauer-Emmett-Teller surface area analyzer (Micromeritics ASAP2020, USA) through nitrogen (99.999%) adsorption-desorption isotherms at -196 °C [19]. Crystallinity index (CrI) was determined from XRD patterns scanned by a powder X-ray diffractometer (X’pert pro, PANalytical, Netherlands) using 40 kV and 40 mA. The diffraction spectra were recorded at 4 ^°^/min in range 2 *θ =* 5 ^°^ to 40 ^°^using a step size of 0.02 ^°^ [20].

1 $$T = {T_m}KC/{\text{ }}\left( {1 + KC} \right)$$

where T is the concentration of adsorbed dye on solid substrate (mg/g); Tm is the concentration of adsorbed dye (mg/g); K is the partition coefficient, and C is the concentration of free dye in the supernatant (g/L).

### Laccase extraction

After incubation for 14 d, fermented substrates were collected and weighted. Meanwhile, precise 1 g of fresh fermented substrate was suspended in 20 ml sodium citrate buffer (0.05 M, pH 4.8) for 12 h to obtain crude laccase solution. After drying at 105 °C to constant weight, a subset of fermented substrate was measured gravimetrically to calculate water content.

### Laccase activity assay

The crude laccase solution was centrifugated at 6200×g for 10 min. And the laccase activity of supernatant was assayed by oxidation of ABTS in sodium citrate buffer (50 mM, pH 3.5) at around 25 °C through monitoring the A_420_ variation spectrophotometrically. One unit (U) of enzyme activity was defined as the amount of laccase required oxidizing 1 µmol of ABTS per minute according to the reported procedure [21]. Laccase production was expressed as U per g dry residue (U/g). The laccase was also purified as the procedure described by Si [22] and assayed by Western blot (Supplementary data 1).

### Measurement of dry material loss during solid state fermentation

The mass and composition loss after SSF was calculated with the Eq. 2. and Eq. 3, respectively.

2 $${Y_T}\left( \% \right) = {\text{ }}\left( {{M_i} - {M_f}} \right)/{M_f} \times 100$$

Where, Y_T_ was the total mass loss after SSF for 14 days, M_i_ and M_f_ was the initial and final dry weight, respectively.

3 $${Y_c}\left( \% \right){\text{ }} = {\text{ }}({X_i} - {X_f} \times {Y_T})/{M_f} \times 100$$

Where, Y_C_ was the composition loss after SSF, X_i_ and X_f_ was the initial and final composition content in samples, respectively.

### Sectioning, staining, antibody labeling and imaging

Microstructure of unfermented solid substrates was observed by fluorescent microscope (ZEISS Axio Scope A1, Germany) with a GFP brightline filter cube. The fresh fermented substrates were directly observed by stereomicroscopy (Leica M165C, USA). In parallel, a subset of fresh fermented substrates was embedded using tissue freezing medium under − 20 °C and sectioned by cryostat (Leica RM2015, USA) at thickness of 8 μm. The randomly selected lengthways slices were labeled using aniline blue solution (1%, w/v) at 70 °C for 10 min, rinsed three times by phosphate-buffered saline (PBS, pH 7.4) for 10 min, and further examined by optical microscope (Teelen TL2800A, China). The other lengthways slices were blocked with PBS containing 3% non-fat milk for 30 min and washed three times with PBS for 5 min. The blocked lengthways slices were then incubated for 1 h with primary antibody for laccase diluted 1:100 in PBS and a secondary antibody diluted 1:200 in PBS, in sequence [23]. After each incubation, the lengthways slices were washed three times with PBS for 10 min and further examined by confocal laser scanning microscopy (CLSM, NIKON AIR, Japan) with the identical instrument settings: 10 × /1.40 NA Plan-Apochromatic objective lens, pinhole size of 0.7 AU, excitation wavelength of 488 nm and emission wavelength of 500–550 nm (green fluorescence) [24]. Immunolabeling of mycelium loaded onto a glass slide were performed as the same procedure. Negative immunolabeling of mycelium or solid substrates was also performed as the same procedure, yet, using only primary antibody or secondary antibody.

### Statistical design

All data in this work were the average value from original triplications and showed as the mean ± standard deviation. The statistical test using *Tukey’s tests* by SPSS (IBM version 16, USA) was performed to confirm the difference between the means.

## Results and discussion

### Preparation of solid substrates by sodium hydroxide pretreatment

#### Physicochemical characteristics of solid substrates

The physical and chemical properties of solid substrates after pretreatment were summarized in Table 1. Additionally, chemical composition of rice straw before and after the pretreatment was given in Table S1. The holocellulose content increased from 63.83% (CK) to 81.47% (PSS) after pretreatment of 0.5 h and exhibited stably with prolonged time. Alkaline pretreatment at this temperature has little effect on hemicellulose degradation; the increase in holocellulose content mainly attributes to the removal of lignin and other alkaline soluble substance [25]. Although cellulosic carbon for enzyme production is beneficial to cost reduction, cellulose in lignocellulosic substrate is masked by hemicelluloses and lignin, which lead to a complex and rigid structure restricting utilization. Novy points out that deconstruction of lignin recalcitrance highly positively correlates with the fungi growth and enzyme production [26]. Sousa also reports that more hemicelluloses in solid substrates can not only provide sufficient carbon but also facilitate mycelium branching and extension [27]. It is said that carbon nutrition roughly takes up 40 ~ 50% of the gross cost in enzyme production [28]. Thus, high preservation of holocellulose in solid substrates was the critical potential for carbon supply.

**Table 1:** Physicochemical characteristics of solid substrates

Substrate	Holocellulose (%)	CrI (%)	CA (mg/g)	SSA (m^2^/g)	ED (mg/g)	WRV (g/g)
CK	63.83 ± 2.60 ^a^	41.02%±0.81^a^	175.44 (*R*^2^ = 0.9951)	1.24 ± 0.19^a^	128.03 ± 1.52^a^	0.71 ± 0.05^a^
PSS (0.5 h)	81.47 ± 0.12 ^b^	39.51%±1.24^ab^	212.77 (*R*^2^ = 0.9509)	1.78 ± 0.09^ab^	258.44 ± 7.35^b^	1.96 ± 0.07^b^
PSS (1 h)	82.58 ± 0.1 ^b^	36.97%±0.30^bc^	434.78 (*R*^2^ = 0.9941)	2.45 ± 0.02^b^	338.60 ± 3.38^c^	2.87 ± 0.41^c^
PSS (1.5 h)	83.63 ± 0.29 ^b^	35.99%±1.70^c^	666.67 (*R*^2^ = 0.9908)	6.85 ± 0.45^c^	375.29 ± 1.99^d^	3.47 ± 0.01^d^

The data labeled by the different superscripts (a, b, c and d) within the same column were different from each other (*p* < 0.05).

Accessibility of solid substrate influences the availability of nutrition and attachment, which is usually assigned to be of two types: micro- and macro-accessibility [29]. The former is impacted by structural properties of cellulose e.g., CrI and CA, whereas, the latter is influenced by porous characteristics e.g., SSA. The CrI decreased from 41.02 to 35.99%, whereas, CA increased from 174.44 to 666.67 mg/g obviously after pretreatment. The fine structure of cellulose is a simplified two-phase model including extremely ordered crystalline parts and less well-ordered amorphous parts. The crystalline parts are major challenges for glucose utilization [30]. The CrI depicts the relative amount of crystalline component compared to the amorphous component in cellulose. The decrease in CrI indicates the rupture of hydrogen bonds in cellulose chains and deconstruction of crystalline region. Hassan shows that enzyme production is inverse proportional to CrI, yet, positive to hemicellulose content [31]. The increase in CA indicated the expose of cellulose, which could be attributed to the deformation in rigid matrix of hemicelluloses and lignin caused by the lignin removal. Additionally, the SSA increased from 1.24 to 6.85 m^2^/g obviously. Our previous study reports that higher SSA facilitates the attachment of mycelium on solid substrates and is beneficial for the interior nutrition utilization [32]. Hence, pretreatment improved the availability of lignocellulosic carbon resource, which was also supported by the desirable in vitro ED. Concretely, the maximum ED was from the PSS (1.5 h), which was 193.13% times higher than that of the CK. Higher in vitro ED indicated that hyphae could penetrate the solid substrate and utilize the carbon resource more easily [33].

Inadequate moisture always inhibits fermentation performance due to the comparatively hydrophobic property of lignocellulosic substrate [34]. Lower WRV restricts nutrient diffusion, reduces swelling and diminishes oxygen transfer, whereas, higher WRV indicates the preferably water retention providing enough water for microbial growth, especially in the latter stage of SSF [35]. WRV of the PSS were observably all higher than that of the CK, which increased by 176.05%, 304.23% and 388.73%, respectively. It is reported that solid to liquid ratio of around 1:3.5 (w/v) is the optimum moisture content for enzyme production [28, 36]. Preservation of homocellulose, improved carbon availability and suitable water retention ability determined here were summed to manifest the improved fermentability of solid substrates. As a consequence, sodium hydroxide pretreatment seemed to be an ideal method to optimize the properties of solid substrate.

#### Morphology of solid substrates

**Fig. 1:**
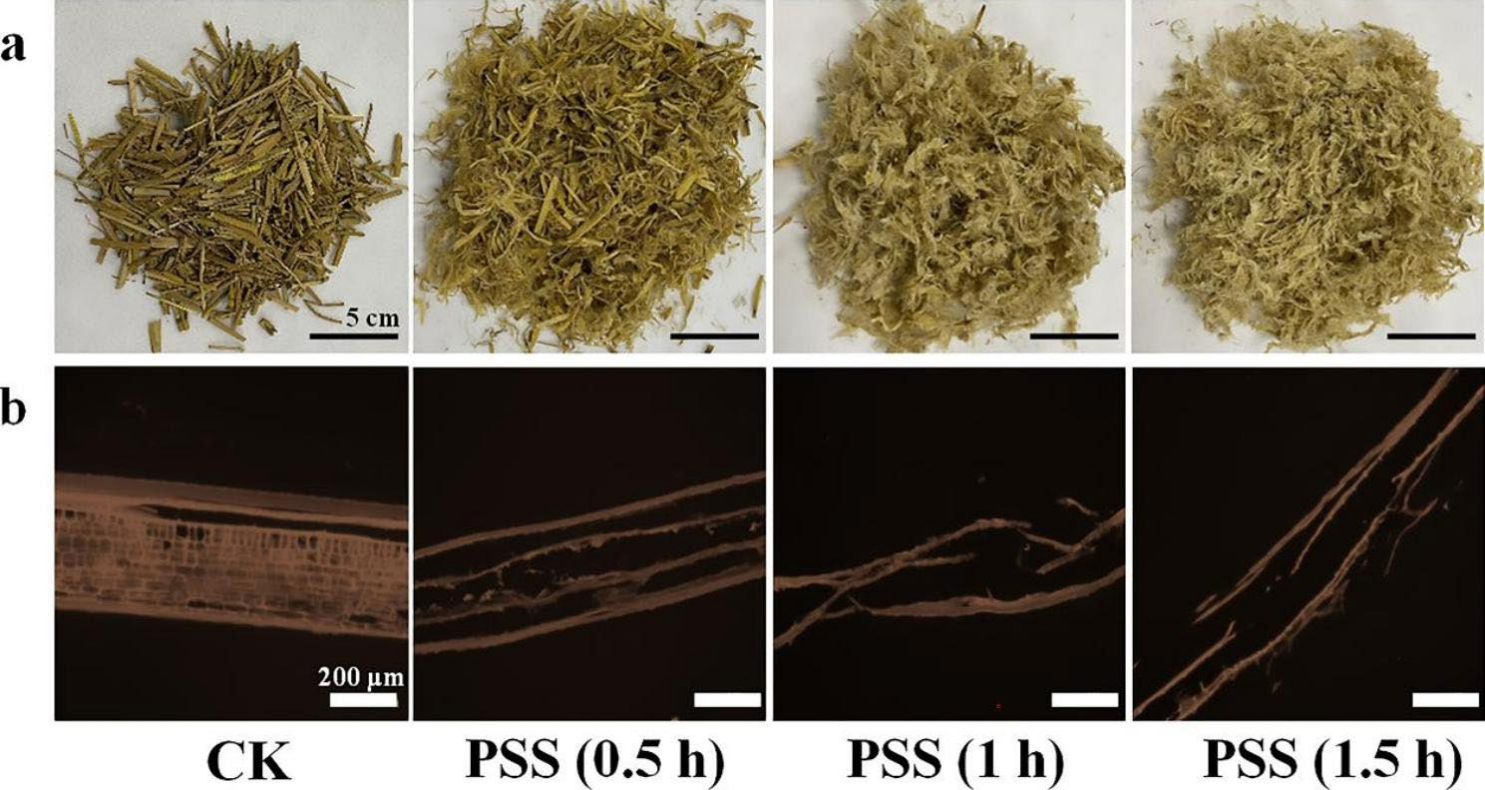
Images showing solid substrates. (a) Photograph for solid substrates. (b) Micrograph for solid substrates by fluorescent microscope. Yellow was lignin autofluorescence

As expected, pretreatment destroyed the rigid structure. The PSS were reduced, whitened from dark yellow and twined after drying. Notably, the surface became visually rough and porous. With the increase in pretreatment time, the PSS became finer and more agglomerated (Fig. 1a). Huang suggests that the brownish yellow change is due to the dissolution of pectin, and lignin [37]. Based on microscopy observation, lengthways view of CK gave the typical tissue structure of monocotyledons containing the outermost epidermis cells covered with cuticle layer, scattered vascular bundles and predominate parenchyma cells (Fig. 1b). After pretreatment, the intact structure of tissue was diminished, and fibers were separated from each other and crushed. The epidermis cells were partially destroyed with space on the edge. As pretreatment proceeded, cell structures were entirely damaged, leaving long and fluffy fibers with indistinguishable structure. Wang reports that NaOH/urea pretreatment can efficiently disrupt the dense epidermis cells and vascular bundles [38], which was similar to our results.

### The impacts of different solid substrates on solid state fermentation

#### Laccase production after solid state fermentation

**Fig. 2:**
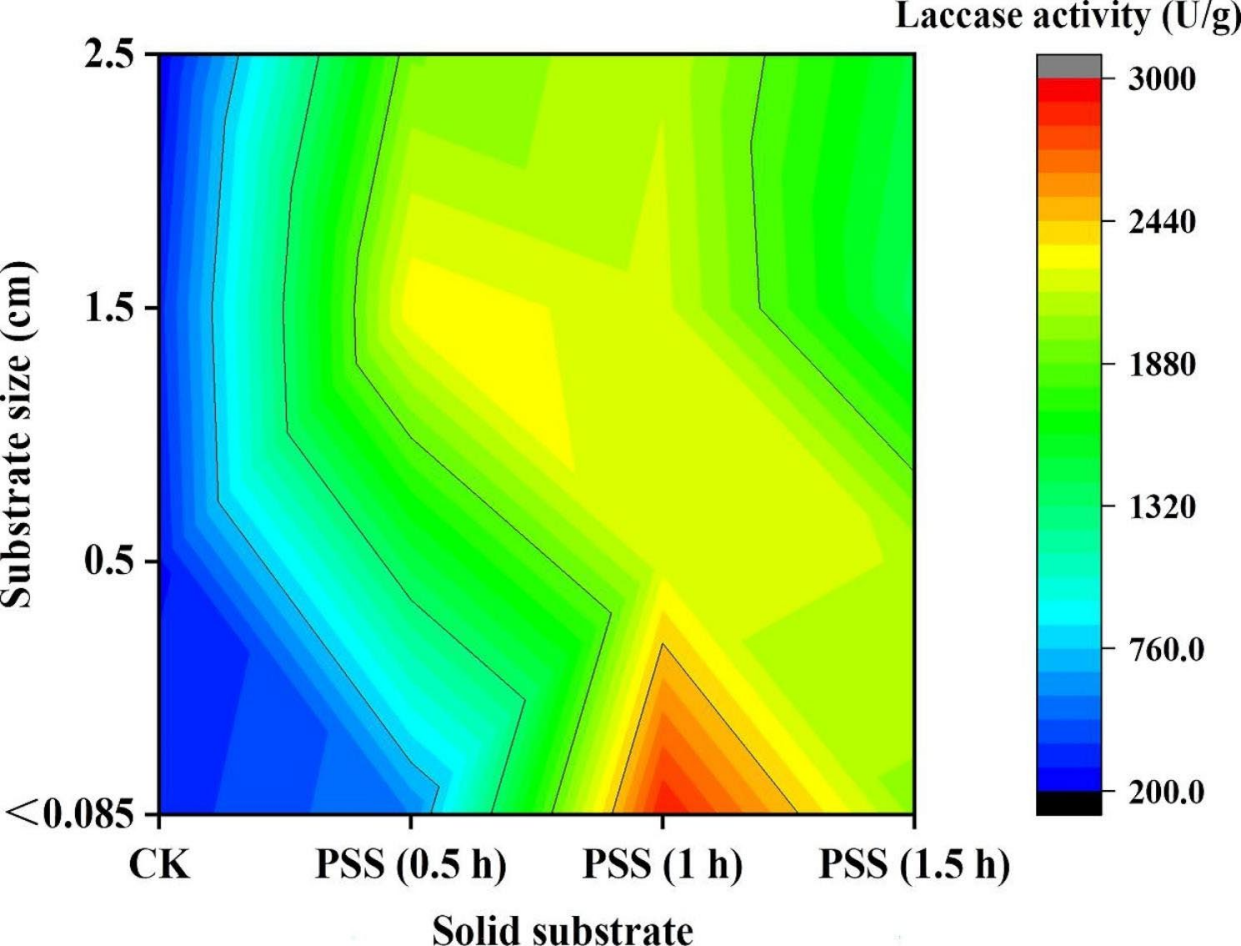
Laccase production contour in SSF with *F. trogii* for 14 d

*F. trogii* has been verified as an interesting candidate for laccase production due to its excellent productivity, inexpensive culture medium and selectable cultivation techniques [21]. Laccase production through SSF using various solid substrates was conducted in glass columns for 14 d. Characteristic band pattern from Western assay verified that *F. trogii* could secrete laccase (Fig. S1). Analytical comparisons showed that the PSS all gave higher laccase production than the CK regardless of substrate size (Fig. 2). In parallel, the optimal solid substrate to improve laccase production was obtained from pretreatment time of 1 h and followed by 0.5 h. Additionally, the smaller substrate size commonly correlated with the higher laccase production. The maximum laccase production (2912.34 U/g) was revealed from the PSS (1 h) with size less than 0.085 cm, which was 7.72 times higher than that of the CK with the similar size.

Substrate size is an obvious aspect impacting the microbial growth and enzyme production [39]. Overall, bigger size provides limiting surface area, whereas, smaller size gives larger surface area required for fungi action. Interestingly, positive influences of milled substrates on laccase production became ambiguous with respect to the PSS. Because substrate size whether higher or lower side adversely impacts SSF. Pretreatment improves the accessibility facilitating the mycelium growth, but, smaller size or higher accessibility also results in substrate agglomeration and channeling problems. Pretreatment of solid substrate has a long history in SSF e.g., high-temperature cooking and smashing, accommodating both enzyme production and downstream processing [40]. Shinkawa selects the ammonia treated rice straw as solid substrate for saccharification enzyme production by the recombinant *Aspergillus oryzae* [41]. Chen selects the steam exploded corn straw as solid substrate for cellulase production by *T. reesei* [42]. Singh selects the microwave coupled with sodium hydroxide treated rice straw, saw dust, and corn straw as solid substrate for xylanase production by *A. flavus* [43]. Salim selects ultrasound treated wheat bran as solid substrate for pectinase production by the *Bacillus. thurgiensis* [44]. Although a direct comparison of the enzyme production reported in various studies cannot be made because no standard SSF process have been adopted, all these researches demonstrate that pretreatment of lignocellulosic waste is beneficial for hydrolytic enzyme production through SSF (Table 2). Among these, alkaline pretreatment seemed to be an effective and relatively simple process scheme, conceivably enhancing oxidase production.

**Table 2:** Comparison of various pretreatment methods used in SSF.

Solid substrate	Pretreatment	Species	Products	Improved efficiency (times)	Reference
Rice straw	Sodium hydroxide pretreatment (4%, 70 °C, 1 h)	*F. trogii*	Laccase	7.72	This research
Rice straw	Aqueous ammonia pretreatment (25%, 80 °C, 8 h)	*A. oryzae*	Cellulase	4.7	[41]
Corn straw	Steam explosion pretreatment (1.5 MPa, 5 min)	*Trichoderma* *Reesei*	Cellulase	4.56	[42]
Rice straw	Microwave with sodium hydroxide pretreatment (2%, 10 min)	*Aspergillus flavus*	Xylanase	2	[43]
Wheat bran	Ultrasound pretreatment (amplitude 20%, 5 min)	*B. thurgiensis*	Pectinase	3.71	[44]

#### Mass loss after solid state fermentation

**Fig. 3:**
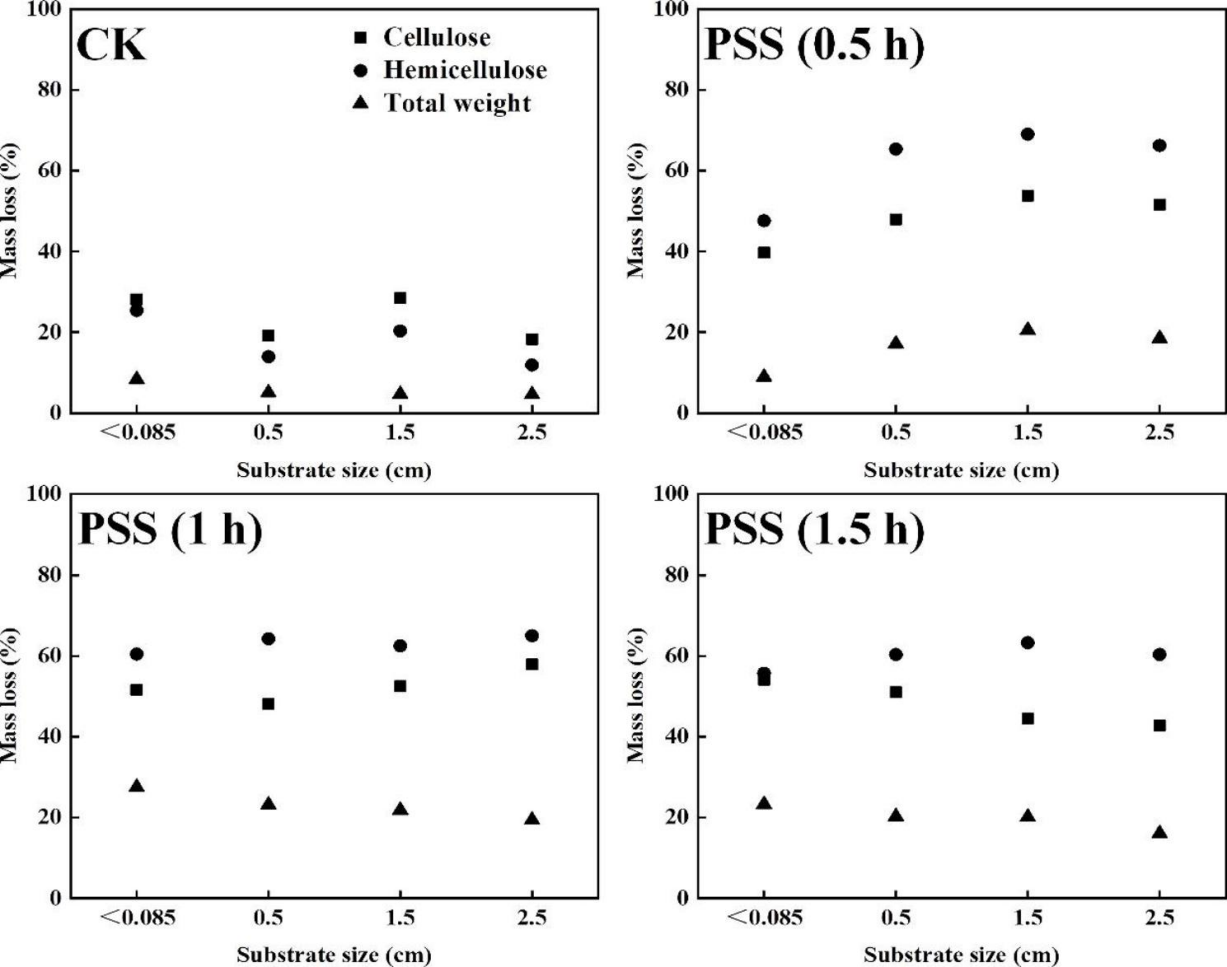
Mass loss after SSF with *F. trogii* for 14 d

The general degradation patterns of PSS by *F. trogii* were almost identical (Fig. 3). Mass loss of PSS was higher than CK, which positively correlated with laccase production. Utilization of cellulose and hemicelluloses in PSS was around 50% and 60%, respectively. This was in line with the aforementioned presumption that preservation of hemicelluloses and cellulose would be beneficial for SSF. Interestingly, it seemed that 81.47% of holocellulose might be enough for *F. trogii*, meanwhile, pretreated hemicelluloses were more easily consumed rather than cellulose. Fungi are complex multicellular structure and possess an inherent inclination to adhesion on substrate as compared with bacteria. The excess substrate degradation causes the collapse of supporting framework, which further impedes heat and mass transfer in SSF [45]. Thus, the reasonable substrate utilization might be an important parameter impacting fermentation performance, which should be studied furthermore.

### The interaction between solid substrate and fungi

**Fig. 4:**
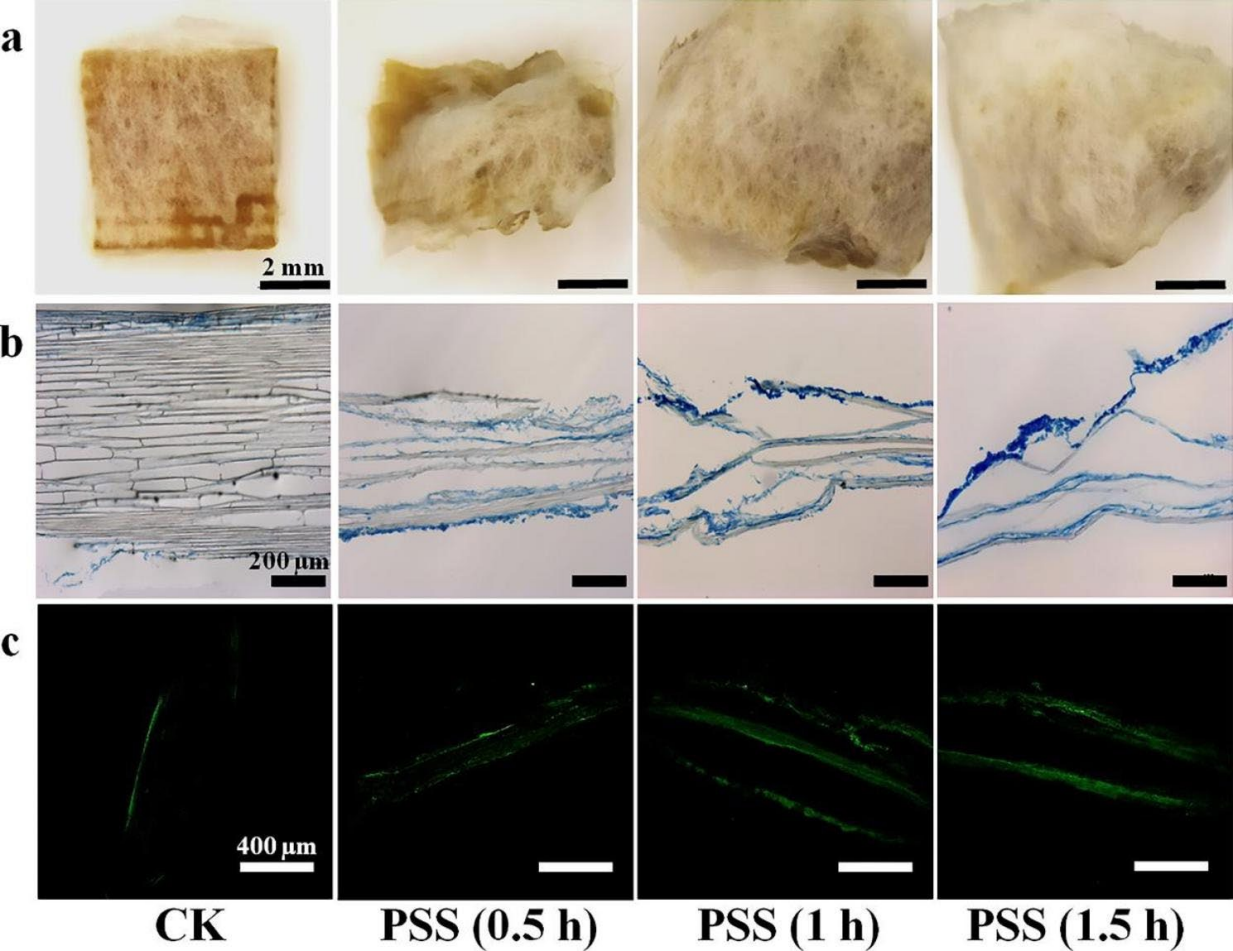
Images showing distribution of laccase and mycelium on solid substrates after SSF with *F. trogii* for 14 d. (a) Photograph for mycelium on solid substrates by stereomicroscopy. (b) Micrograph for mycelium on solid substrates by optical microscope. (c) Micrograph for laccase on solid substrates by CLSM.

After incubation, only tiny mycelium attached to surface of the CK, yet, apparently denser mycelium growth was confirmed on the PSS (Fig. 4a). This is in accordance with the facts that fungal mycelium in natural habitat tend to penetrate, elongate and branch in the solid substrate [46]. Solid substrates were coagulated with one other by visual inspection, especially for the PSS. Staining analysis suggested that mycelium only grew on outer surface of the CK, indicating that epidermis cells impeded penetration of *F. trogii* to inner space (Fig. 4b). After pretreatment, mycelium could grow into the inner and intertwine with substrate. Continuous growth and uniform distribution of mycelium can stimulate enzyme production [47]. The laccase release was also monitored by CLSM. Green fluorescence was captured throughout the cell membrane, whereas, no green fluorescence of negative immunolabeling was observed, suggesting that antibody can specifically adsorb with laccase (Fig. S2). Antibody labeling analysis demonstrated that only little laccase was observed on the outer epidermis cells of the CK (Fig. 4c). In parallel, large amount of laccase were distributed uniformly in the cracks and inner of the PSS. Thus, pretreatment of solid substrate enabled the production and distribution uniformity of laccase, which was consistent to the former results.

**Fig. 5:**
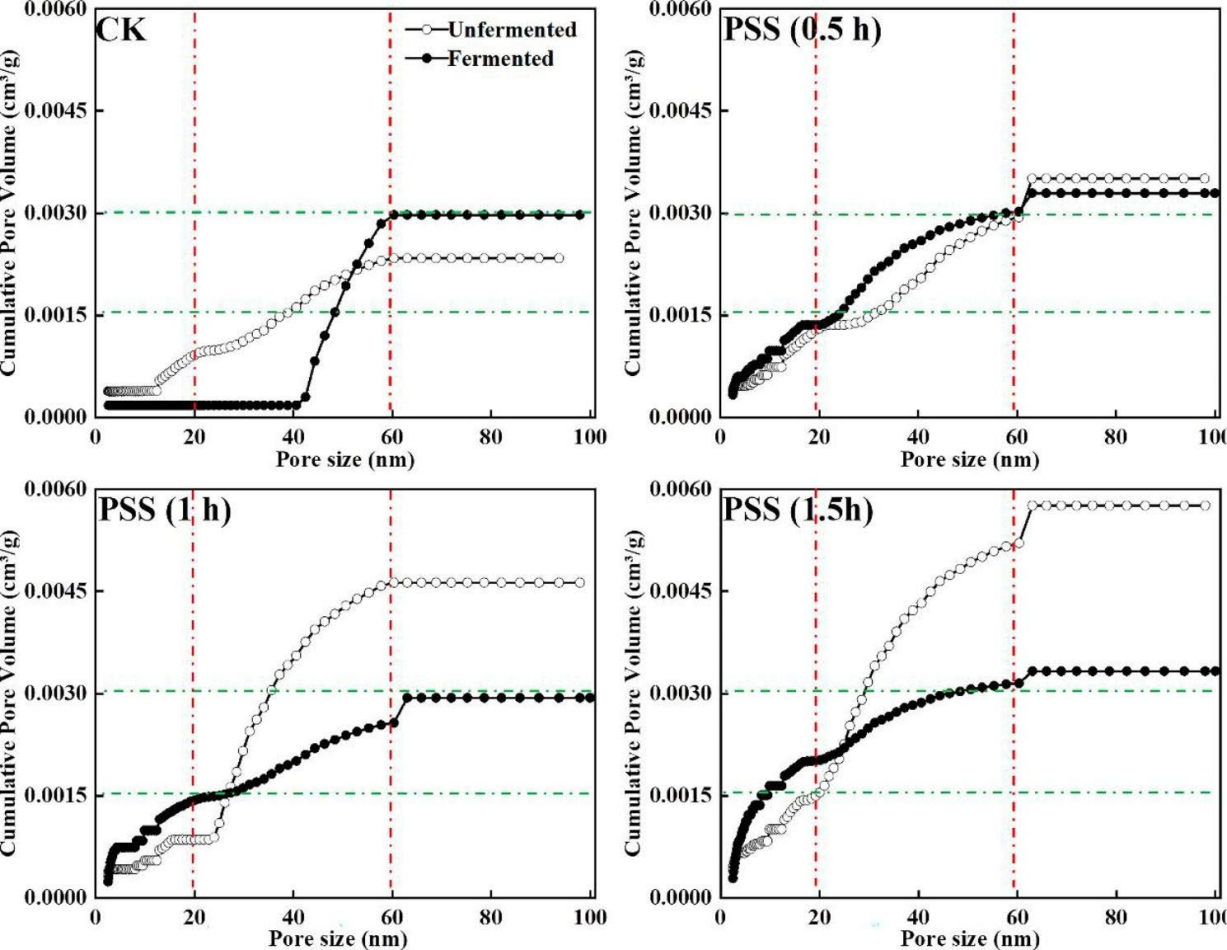
Pore size distribution of solid substrates after SSF with *F. trogii* for 14 d

Pore size ranging from 0 to 100 nm distributions of fermented and unfermented solid substrates were determined and shown in Fig. 5. For concrete pore, higher pore volume suggested the more pore number and vice versa. Fewer pores were detected in the CK, which was deemed as one of the reasons relating to the low efficiency in SSF [48]. The pore volume augmented obviously after pretreatment, particularly for the pore diameter ranging from 20 to 60 nm, suggesting that pores with diameter less than 20 nm were transferred into larger pores. This also corroborated the deconstruction of rigid structure. It seemed likely that poor performance of the CK was at least partly attributed to the large number of small pores. After SSF, the volumes were notably decreased and maintained around 0.003 cm^3^/g dry substrate. Additionally, the fermented PSS gave more homogeneous pore structure compared to CK, implying the homogeneity of nutrition utilization. Thus, the effects of solid substrates were not solely due to composition and accessibility, but also were dependent on the structure support e.g., pore features, which were often highly interrelated and difficult to be assessed individually. Therefore, an adequate balance between them was a must for SSF with different requirements and should be investigated in depth.

## Conclusions

Sodium hydroxide pretreatment disrupted the complex recalcitrance of rice straw to prepare solid substrates with high preservation of lignocellulosic carbon source, noticeable accessibility and optimal WRV. SSF using the PSS exhibited higher laccase production and carbon utilization, and homogeneous distribution of mycelium and laccase. In summary, it was undoubtedly concluded that sodium hydroxide pretreatment of lignocellulosic waste showed excellent performance to enhance fermentation efficiency, which might be a promising technique for industry-scale SSF. Additionally, sufficient balance analysis between nutrition accessibility and structure support of solid substrate must be conducted before SSF with various process requirements.

## Electronic supplementary material

Below is the link to the electronic supplementary material.

Supplementary Material 1

## Data Availability

The datasets generated and/or analyzed during the current study are not publicly available due to the authors’ decision but are available from the corresponding author on reasonable request.

## Abbreviations

ABTS: 2, 2-Azinobis (3-ethylbenzo-thiazoline-6-sulfonic acid)
CA: Cellulose accessibility
CK: Blank control
CLSM: Confocal laser scanning microscopy
CrI: Crystallinity index
ED: Enzymatic digestibility
GFP: Green fluorescent protein
PBS: Phosphate-buffered saline
PDM: Polyvinylidene difluoride membrane
PSS: Pretreated solid substrate
SF: Submerged fermentation
SPSS: Statistical Package for the Social Sciences
SSA: Specific surface area
SSF: Solid state fermentation
WRV: Water retention value
XRD: X-ray diffractometer
