# Supplementary material for: The feasibility of sodium hydroxide pretreatment of rice straw for solid substrate preparation to enhance laccase production by solid state fermentation
Source: BMC Biotechnol. 2023 Jun 30;23:16. doi: 10.1186/s12896-023-00789-3 (PMC10314400; doi:10.1186/s12896-023-00789-3)
Supplement: Supplementary file 1 — Supplementary Material 1 [file 12896_2023_789_MOESM1_ESM.docx]

Supplementary data 1: **Fig. S1**. Western blot assay.


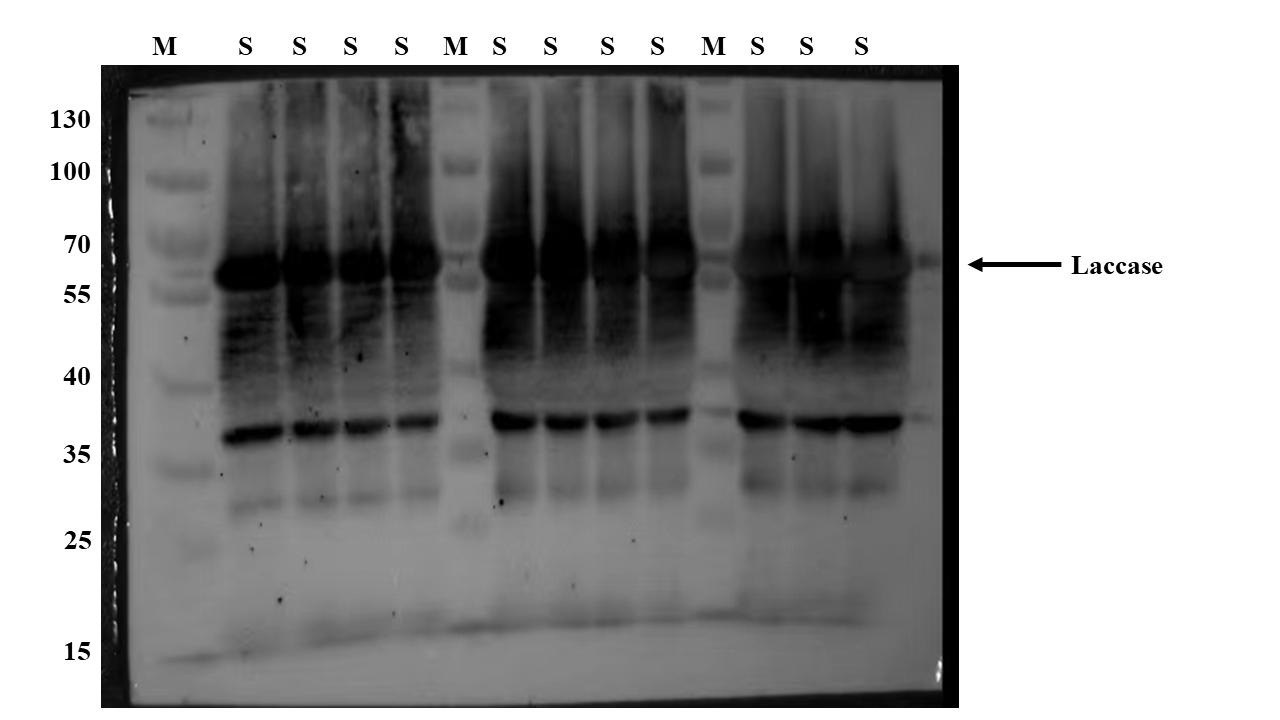


M: marker; S:laccase.

Supplementary data 2: **Fig. S2**. Negative immunolabeling of mycelium.


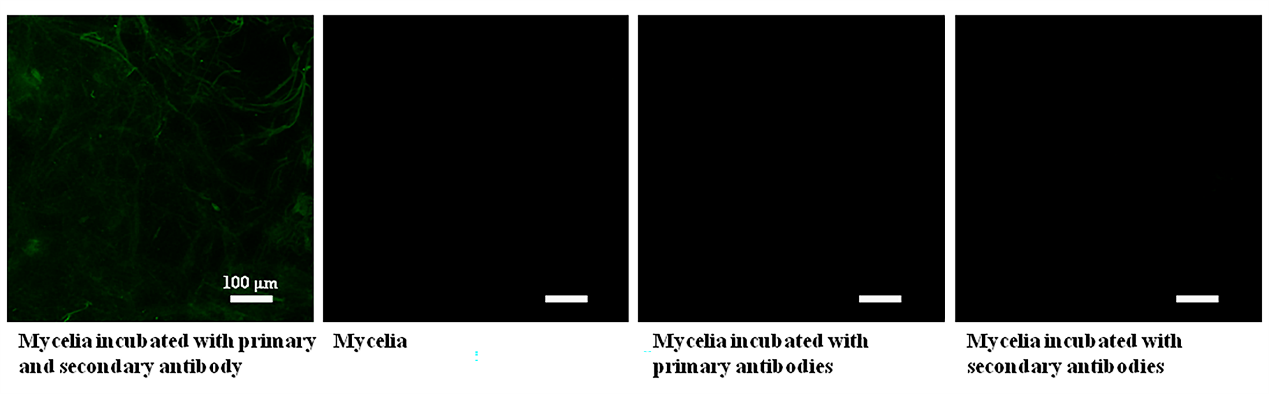


Supplementary data 3: **Fig. S3.** Negative immunolabeling of solid substrates.


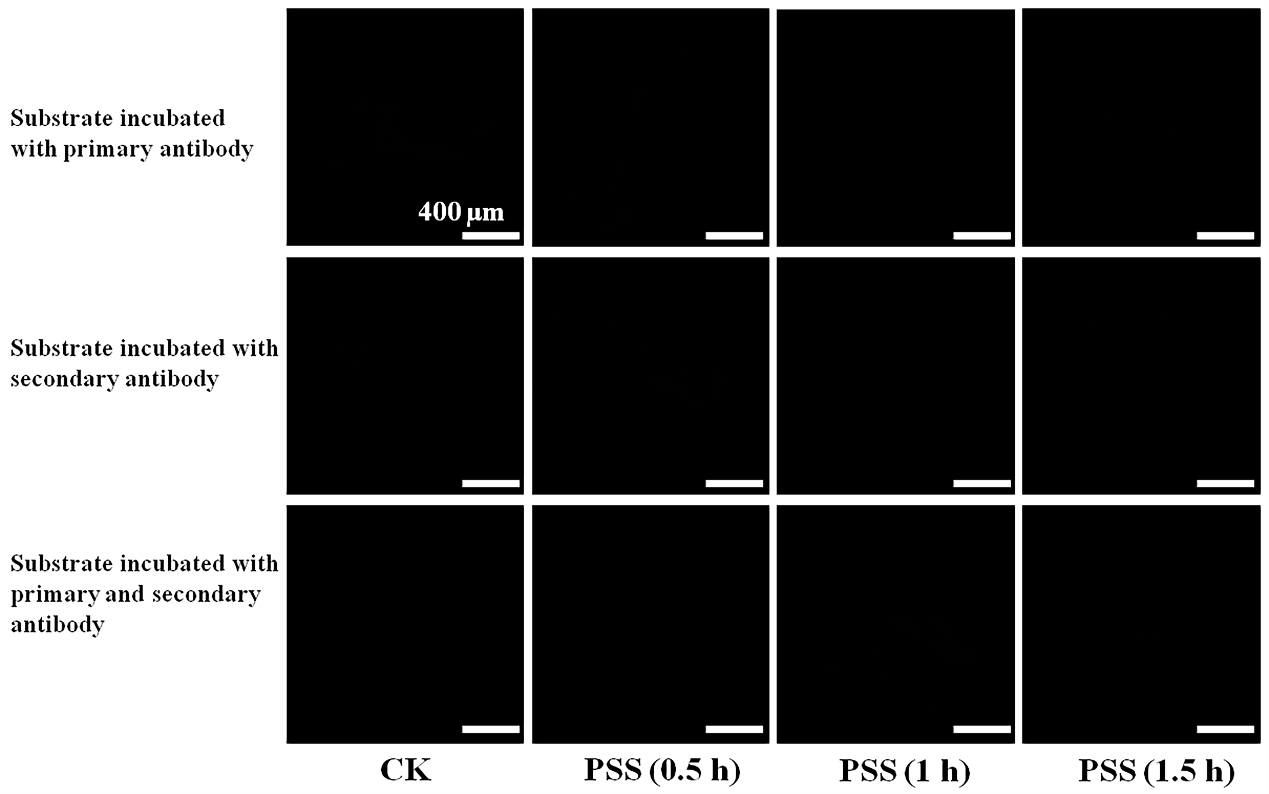


Supplementary data 4: Table S1 Chemical composition of rice straw before and after the pretreatment.

| Substrate | Neutral detergent soluble (%) | Cellulose (%) | Hemicellulose (%) | Lignin (%) |
| --- | --- | --- | --- | --- |
| CK | 76.31±2.30^a^ | 36.24±2.31^a^ | 27.42±0.29^a^ | 12.47±0.31^a^ |
| PSS (0.5 h) | 84.84±0.05^b^ | 61.46±0.00^b^ | 20.01±0.12^b^ | 3.37±0.17^b^ |
| PSS (1 h) | 85.51±0.01^b^ | 64.24±0.05^bc^ | 18.34±0.15^c^ | 2.92±0.09^b^ |
| PSS (1.5 h) | 85.89±0.27^b^ | 66.30±0.67^c^ | 17.33±0.38^d^ | 2.26±0.02^c^ |

The data labeled by the different superscripts (a, b, c and d) within the same column were different from each other (*p*<0.05).
